# The impact of screening-detected atrial fibrillation and associated outcomes on quality of life

**DOI:** 10.1007/s11136-025-04047-1

**Published:** 2025-08-31

**Authors:** Emilie Katrine Kongebro, Christian Kronborg, Ketil Jørgen Haugan, Claus Graff, Søren Højberg, Derk Krieger, Axel Brandes, Lars Køber, Jesper Hastrup Svendsen, Søren Zöga Diederichsen

**Affiliations:** 1 Department of Cardiology, Copenhagen University Hospital – Rigshospitalet, Inge Lehmanns Vej 7, Copenhagen, 2100 Denmark; 2 Department of Economics, University of Southern Denmark, Campusvej 55, Odense, 5230 Denmark; 3 Department of Cardiology, Zealand University Hospital Roskilde, Sygehusvej 10, Roskilde, 4000 Denmark; 4 Department of Health Science and Technology, Aalborg University, Selma Lagerløfs Vej 249, Gistrup, 9260 Denmark; 5 Department of Cardiology, Bispebjerg Hospital, Copenhagen University Hospital, Bispebjerg Bakke 23, Copenhagen, 2400 Denmark; 6 Mohammed Bin Rashid University of Medicine and Health Sciences, Al Razi St - Umm Hurair 2 - Dubai Healthcare City, Dubai, UAE; 7 Department of Neurology, Mediclinic Parkview Hospital, Umm Suqeim St - Arjan-Dubailand - Al Barsha South, Dubai, UAE; 8 Department of Regional Health Research, Faculty of Health Sciences, University of Southern Denmark, Finsensgade 35, Esbjerg, 6700 Denmark; 9 Department of Cardiology, Esbjerg Hospital – University Hospital of Southern Denmark, Finsensgade 35, Esbjerg, 6700 Denmark; 10 Department of Clinical Medicine, Faculty of Health and Medical Sciences, University of Copenhagen, Blegdamsvej 3B, Copenhagen, 2200 Denmark

**Keywords:** Atrial fibrillation, Population-based Screening, Quality of Life, Stroke Prevention, Implantable Loop Recorder, Randomised Controlled Trial

## Abstract

**Purpose:**

To investigate health-related quality of life (HRQoL) after atrial fibrillation (AF) detected by screening compared with conventional AF diagnosis.

**Methods:**

We used HRQoL data (EQ-5D-5L) from 6004 persons randomised to AF screening with implantable loop recorder and treatment (*n* = 1501) or to usual care (*n* = 4503). Annual assessments yielded individual EQ-5D-5L-index (worst=-0.76 best = 1.00) and EQ-VAS scores (Visual Analogue Scale, 0 = worst, 100 = best). Changes were estimated with linear mixed models from before to after incident AF, stroke, and major bleeding. Interaction analyses assessed differences between the randomisation groups.

**Results:**

During three years of follow-up, 693 of 6004 (12%) participants were diagnosed with AF (Screening: 424 of 1501 (28%), usual care: 269 of 4503 (6.0%)), with 636 alive at year three. For participants developing AF, the EQ-5D-5L index score in the screening group declined from 0.87 before to 0.85 after AF (*p* < 0.001), and from 0.83 before to 0.79 after AF (*p* < 0.001) in usual care, with less HRQoL decline in the screening group (*p* = 0.019). For patients developing stroke and major bleeding, the EQ-5D-5L index scores in the screening group declined from 0.82 to 0.78 (*p* < 0.001) and 0.82 to 0.76 (*p* < 0.001) before and after diagnosis, and from 0.84 to 0.76 (*p* < 0.001) and 0.85 to 0.76 (*p* < 0.001) in usual care, without differences between the randomisation groups. All EQ-VAS analyses yielded very similar results.

**Conclusion:**

AF detected through screening had little negative impact on HRQoL compared with AF detected by usual care. Stroke and major bleeding were followed by large HRQoL reductions, regardless of randomisation group.

**Trial registration:**

The LOOP study is registered at ClinicalTrials.gov, identifier: NCT02036450.

**Graphical Abstract:**

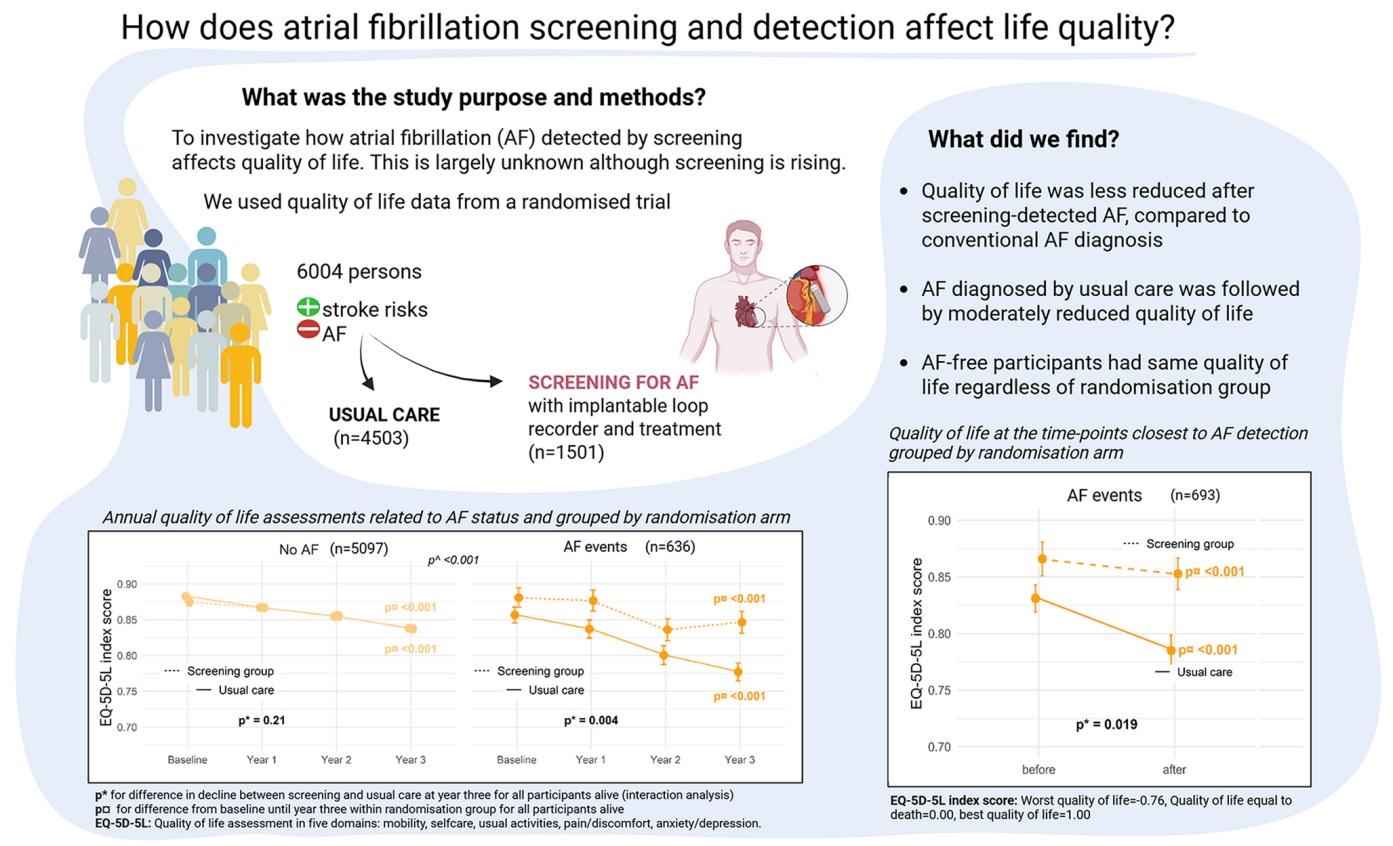

**Supplementary Information:**

The online version contains supplementary material available at 10.1007/s11136-025-04047-1.

## Introduction

Stroke represents a major global health challenge, as it affects one in four persons during their lifetime [1]. At the same time, a substantial number of strokes are attributed to undetected and untreated atrial fibrillation (AF) [2]. This has encouraged several trials to attempt screening for early AF with subsequent treatment, in order to mitigate the morbidity and mortality associated with stroke. A screening strategy should result in an overall net benefit for the patients, weighing the beneficial effects of diagnosis and treatment against potential harms. While screening for other diseases has been associated with both harmful and beneficial effects on HRQoL [3–6], the impact of AF screening has been investigated sparsely. Current evidence has found that screening can increase AF diagnoses [7], but efficacy for stroke prevention is lacking [7, 8]. The harmful effects may in turn include complications such as bleeding and other side effects of medications, psychological impact, and overdiagnosis and overtreatment of clinically irrelevant findings [9]. Health-related quality of life (HRQoL) is used to provide a joint description of several physical and psychological health aspects. It is often considered a less accessible outcome compared with hard outcomes [10], but remains important for patients and healthcare systems [11]. If present, screening-associated adverse effects on HRQoL should be discussed with patients alongside other risks. This study aimed to investigate the impact on HRQoL associated with AF diagnoses and clinical events in patients undergoing AF screening compared with usual care.

## Methods

### Study design

This was a post-hoc analysis of the LOOP study including the full study population. The LOOP study was an investigator-initiated, randomised, controlled, multi-centre trial investigating whether AF screening could reduce stroke in high-risk individuals. Briefly, participants were 70–90 years old, without known AF, but diagnosed with at least one known risk factor for stroke: hypertension, diabetes, previous stroke, or heart failure [12]. Participants were identified via the Danish health registries and invited by letter. Eligible persons answered the baseline quality of life questionnaires and were subsequently randomised in a one-to-three ratio to implantable loop recorder (ILR) screening and initiation of anticoagulation upon detection of AF episodes lasting at least 6 min (screening group) or usual care (control group). HRQoL was assessed from baseline to year three by the EuroQol 5D-5L questionnaire (EQ-5D-5L) [13], and was conducted on-site in the screening group annually, whereas the control group was assessed on-site at baseline and year three and by letter in year one and two. AF diagnoses were adjudicated by cardiologists. Clinical outcomes were captured by annual follow-up visits or remote contacts in addition to look-up in medical records [12]. Major bleeding was defined according to the ISTH criteria. Stroke, transient ischemic attack (TIA) and systemic arterial embolism (SAE) were adjudicated by the clinical endpoint committee in the study [7, 14]. 

### HRQoL data

The EQ-5D-5L questionnaire is a validated and widely used assessment tool for HRQoL, including five separate domains (mobility, selfcare, usual activities, pain/discomfort, and anxiety/depression) and accompanied by the EuroQol visual analogue scale (EQ-VAS). The EQ-5D-5L domains are each scored on a five-level scale ranging from 1 to 5, with 1 translating to “no problem” and 5 referring to an “extreme problem”. All domain scores were converted to a continuous scale using validated and country-specific weights, rendering one EQ-5D-5L index score for every participant [13]. The Danish index (-0.76 to 1.00) is anchored at 1, which is equivalent to full health (i.e. no problems in any domain), and 0, which is equivalent to being dead, while negative values are equal to a health worse than death [13]. The EQ-VAS is reported with the EQ index and reflects participants’ self-reported health in the range 0-100 (0 = worst imaginable health, 100 = best imaginable health).

### Outcomes and statistical analyses

Outcomes were EQ-5D-5L index and EQ-VAS score changes from the closest evaluation before a clinical event to the closest evaluation after a clinical event. Here, the clinical events were AF diagnosis, stroke, major bleeding, and a combined outcome of any of these events. In secondary analyses, the score changes were analysed in participants alive from baseline to the three-year assessment. Events were also grouped by the year of diagnosis (year one, two or three), including HRQoL assessments preceding or following the diagnosis to study the timing of diagnosis and the duration of HRQoL changes. Lastly, we analysed participants free of clinical events from baseline to year three. In relation to the EQ-5D-5L domains, the following categories were applied: any problem = level 2–5 in any EQ-5D-5L domain, major problem = level 3–5 in any EQ-5D-5L domain, and worsening problem = level increments in any EQ-5D-5L domain. Subgroup analyses were based on prior stroke status, sex, and age below or above the median.

Baseline characteristics were presented as frequencies with percentages for categorical variables (compared by chi-squared tests), mean values ± standard deviations for continuous variables with a normal distribution (t-tests), or medians with first and third quartiles for continuous variables with non-normal distributions (Wilcoxon rank-sum tests). Linear mixed model estimates and Odds ratio (OR) were presented with 95% confidence intervals (CI). We used a linear mixed-effects regression model with an unstructured covariance pattern for repeated measurements to estimate the mean changes in EQ-5D-5L index and EQ-VAS scores for each randomisation group [15]. Four visits/contacts were included with an interaction term between the randomisation groups and visits to allow for a dynamic effect of a screening effect over time. The participants who died before the one-year, two-year, or three-year assessments were not included in the analyses of events occurring during year one, year two and year three, respectively, but were reported separately. Generalised estimating equation models with repeated measurement were used to estimate the OR of major problem. Sensitivity analyses were adjusted for age, sex, prior stroke, systemic arterial embolism (SAE) or transient ischemic attack (TIA), chronic obstructive pulmonary disease (COPD) and heart failure. All statistics were performed in R version 4.3.2 or newer.

## Results

### Participants

A total of 6004 participants were randomised; 1501 to screening and 4503 to usual care. The mean age was 74.7 years and 53% were male. The most common comorbidities were hypertension (91%), diabetes (28%), and previous stroke, TIA or SAE (25%) (Table 1) [7]. At the one-year assessment, 5948 of 6004 (99%) participants were alive, while 5850 (97%) and 5733 (95%) were alive at the assessments in year two and three, respectively. At the three-year assessment, 636 participants were alive and had been diagnosed with AF (Screening: 395 (26%); Control: 241 (5.4%), 144 with incident stroke (Screening: 33 (2.2%) Control: 111 (2.5%), and 99 with major bleeding (Screening: 30 (2.0%) Control: 69 (1.5%)). The numbers of participants alive and annual clinical event rates for AF, stroke and major bleeding are given in Table 2. The HRQoL declined slightly in the entire population from baseline to year three, which has been described previously [16]. 

**Table 1:** Baseline characteristics

		Control (*n* = 4503)	Screening (*n* = 1501)
GENERAL			
Age, years	mean (sd)	74.7 (4.1)	74.7 (4.1)
Male sex		2375 (53.7)	792 (53.8)
Smoking status	Never	1782 (39.6)	597 (39.8)
	Current	417 (9.3)	135 (9.0)
	Previous	2302 (51.1)	769 (51.2)
Alcohol consumption, units/week	mean (sd)	7.2 (8.2)	7.3 (8)
Education	Primary School	747 (17.4)	284 (19.5)
	High School or equivalent	1734 (40.3)	608 (41.8)
	Bachelor’s Degree	1397 (32.5)	416 (28.6)
	Master’s Degree	420 (9.8)	146 (10.0)
MEDICAL HISTORY			
Hypertension		4066 (90.3)	1378 (91.8)
Diabetes		1288 (28.6)	422 (28.1)
Stroke, TIA or SAE		1139 (25.3)	370 (24.7)
Myocardial infarction or CABG		614 (13.6)	177 (11.8)
COPD		330 (7.3)	110 (7.3)
Heart failure		199 (4.4)	67 (4.5)
CHA_2_DS_2_VASc Score	2	588 (13.1)	202 (13.5)
	3	1494 (33.2)	513 (34.2)
	4	1325 (29.4)	419 (27.9)
	≥ 5	1096 (24.3)	367 (24.4)
PRESCRIPTIONS			
Platelet inhibitor		2204 (48.9)	702 (46.8)
Betablocker		1172 (26.0)	354 (23.6)
Diuretics		1511 (33.6)	495 (33.0)
Statin		2621 (58.2)	879 (58.6)
Renin-angiotensin inhibitor		2999 (66.6)	991 (66.0)
Antidiabetics		1129 (25.1)	385 (25.6)

**Abbreviations**: COPD; Chronic obstructive pulmonary disease, TIA; Transient Ischemic attack, SAE; Systemic arterial embolism, CABG; Coronary artery bypass grafting

**Table 2:** Study participants alive and clinical events per year

	Study participants alive *n* (%)		AF		Stroke		Major bleeding	
	Screening *n* (%)	Control *n* (%)	Screening *n* (%)	Control *n* (%)	Screening *n* (%)	Control *n* (%)	Screening *n* (%)	Control *n* (%)
*Baseline*	*1501*	*4503*						
Year one	1486 (99)	4462 (99)	252 (17)	76 (1.7)	13 (0.9)	45 (1.0)	18 (1.2)	20 (0.4)
Year two	1465 (98)	4385 (97)	99 (6.8)	96 (2.2)	12 (0.8)	43 (1.0)	10 (0.7)	33 (0.8)
Year three	1441 (96)	4292 (95)	73 (5.1)	97 (2.3)	12 (0.8)	39 (0.9)	10 (0.7)	27 (0.6)

### HRQoL after AF diagnosis

**Fig. 1:**
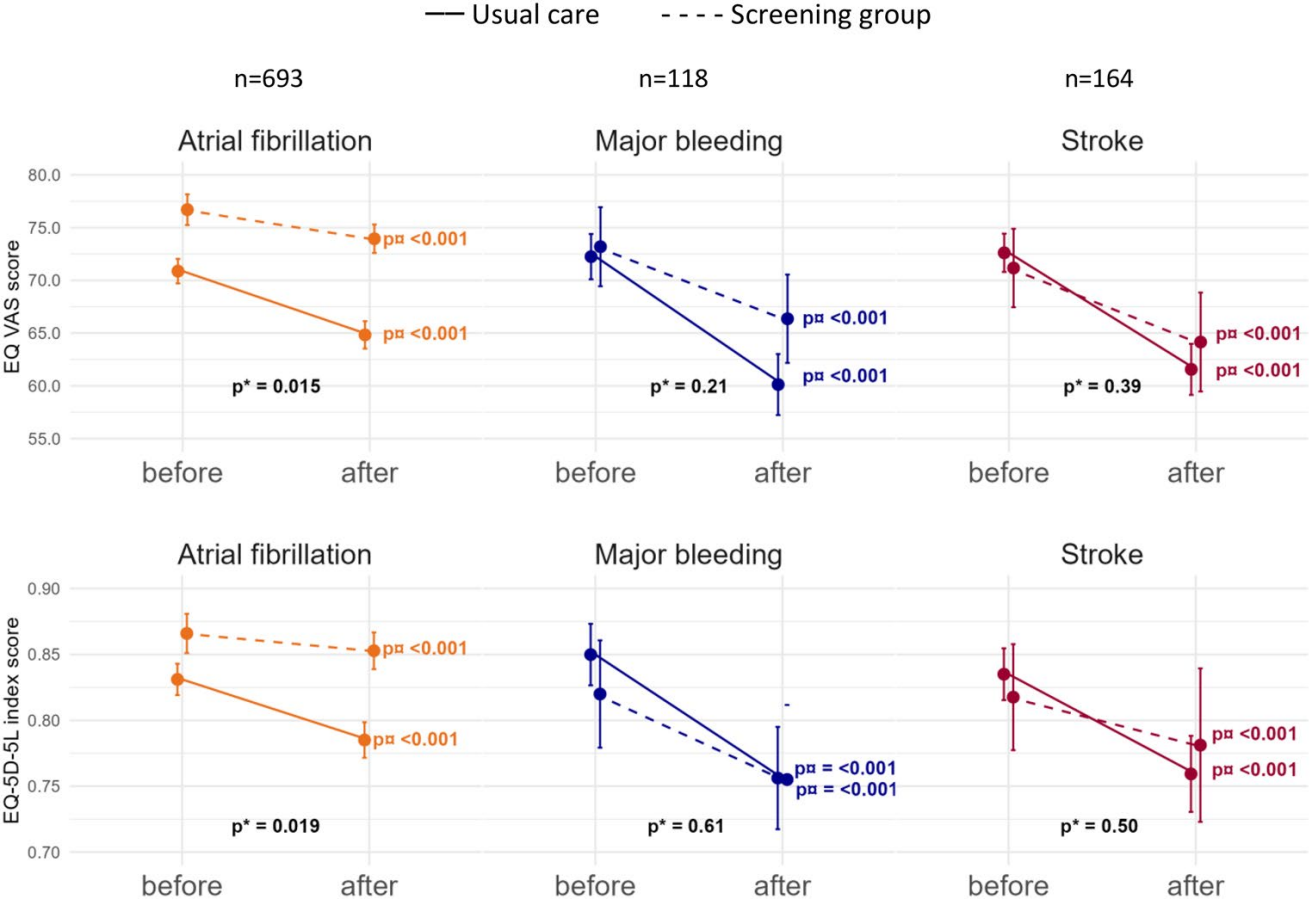
Quality of life before and after AF, stroke and major bleeding. These analyses compare the closest HRQoL before diagnosis with the closest HRQoL after. p* for the difference in decline between screening and usual care (interaction analysis). p¤ for difference in HRQoL from before to after event within randomisation group. For all events, chi squared tests showed no difference in time to event between the randomisation groups

**Fig. 2:**
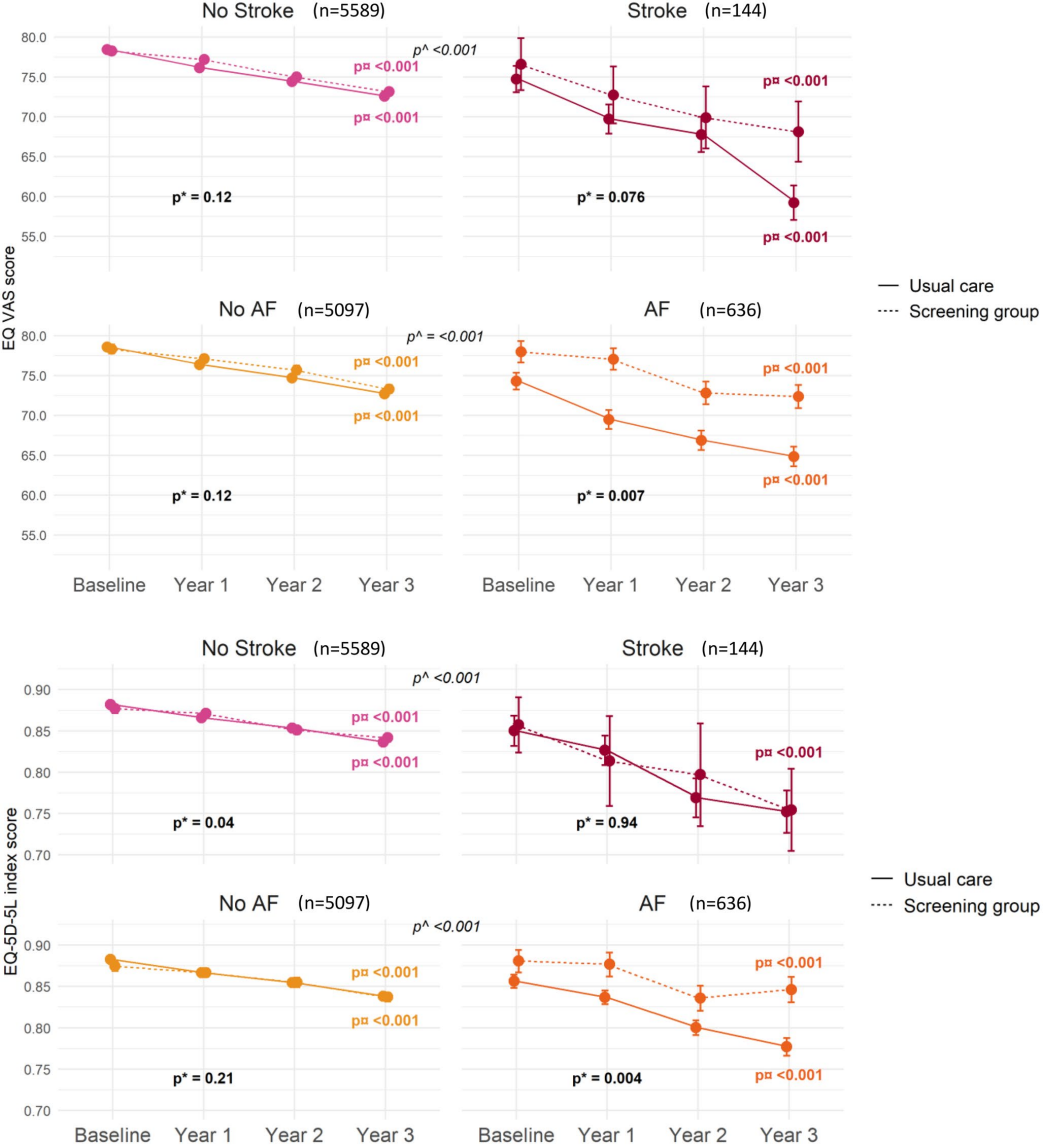
Quality of life associated with AF and stroke detected between baseline and year three. p* for difference in HRQoL decline between screening and usual care at year three (interaction analysis). p¤ for difference in HRQoL from baseline until year three within randomisation group. p^ for difference in HRQoL after event vs. no event

In participants diagnosed with AF, the EQ-VAS score declined from 76.7 before to 73.9 after AF diagnosis (*p* < 0.001) in the screening group and from 70.9 before to 64.8 after AF (*p* < 0.001) in usual care, resulting in less HRQoL decline in the screening group (*p* = 0.015, Fig. 1). The EQ-5D-5L index score declined from 0.87 before to 0.85 after AF (*p* < 0.001) in the screening group and from 0.83 before to 0.79 after AF (*p* < 0.001) in usual care, resulting in less HRQoL decline in the screening group (*p* = 0.019). When looking from baseline to the end of year three, the EQ-VAS score declined from 78.0 to 72.8 (*p* < 0.001) in the screening group and from 74.3 to 64.8 (*p* < 0.001) in usual care, resulting in less HRQoL decline in the screening group (*p* = 0.007, Fig. 2). From baseline to the end of year three, the EQ-5D-5L index score declined from 0.88 to 0.85 (*p* < 0.001) in the screening group and from 0.86 to 0.78 (*p* < 0.001) in usual care, with less HRQoL decline in the screening group (*p* = 0.004). Sensitivity analyses adjusted for age, sex and CHA_2_DS_2_VASc score did not change these findings.

To study the timing of AF detection, participants were grouped by the year of AF diagnosis and followed until the end of year three (Supplementary Fig. S1). The baseline HRQoL values were significantly different between the randomisation groups for AF detected during year one (*p* = 0.002), and for participants diagnosed with AF during year one and two, the EQ-VAS scores were higher in the screening group at the year-one assessment compared to usual care (*p* = 0.01, *p* = 0.018). For participants diagnosed with AF during year two, the EQ-VAS score declined from 77.1 at baseline to 74.5 at the end of year three (*p* < 0.001) in the screening group, compared to a decline from 76.3 at baseline to 67.2 at the end of year three (*p* < 0.001) in usual care, resulting in less HRQoL decline in the screening group (*p* = 0.002). The corresponding EQ-5D-5L index score declined from 0.88 at baseline to 0.86 at the end of year three (*p* < 0.001) in the screening group and from 0.87 at baseline to 0.78 at the end of year three (*p* < 0.001) in usual care, with less HRQoL decline in the screening group (*p* = 0.005).

### HRQoL after stroke, bleeding, or combined outcome

In participants diagnosed with incident stroke, the EQ-VAS score declined from 71.2 before to 64.2 after (*p* < 0.001) stroke in the screening group and from 72.6 before to 61.6 after stroke (*p* < 0.001) in usual care, with no difference in decline between the randomisation groups (*p* = 0.39). The EQ-5D-5L index score declined from 0.82 before to 0.78 after stroke (*p* < 0.001) in the screening group and from 0.84 before to 0.76 after stroke (*p* < 0.001) in usual care, with no difference in decline between the randomisation groups (*p* = 0.57). From baseline to the end of year three, there were no HRQoL differences between randomisation groups in participants diagnosed with stroke (*p* = 0.076 and *p* = 0.94). Adjustment for stroke severity (Modified Ranking Score) at discharge did not alter the estimates, nor did adjustment for participants with AF detected or type of stroke.

In participants diagnosed with major bleeding, the EQ-VAS score declined from 73.2 before to 66.4 after major bleeding (*p* < 0.001) in the screening group and from 72.5 before to 60.1 after major bleeding (*p* < 0.001) in usual care, and the EQ-5D-5L index score declined from 0.82 before to 0.76 after major bleeding (*p* < 0.001) in the screening group and from 0.85 before to 0.76 after major bleeding (*p* < 0.001) in usual care, with no HRQoL differences in decline between the randomisation groups (*p* = 0.21, *p* = 0.67). From baseline to the end of year three, there were no HRQoL differences between randomisation groups in participants diagnosed with major bleeding (*p* = 0.20, *p* = 0.90).

**Fig. 3:**
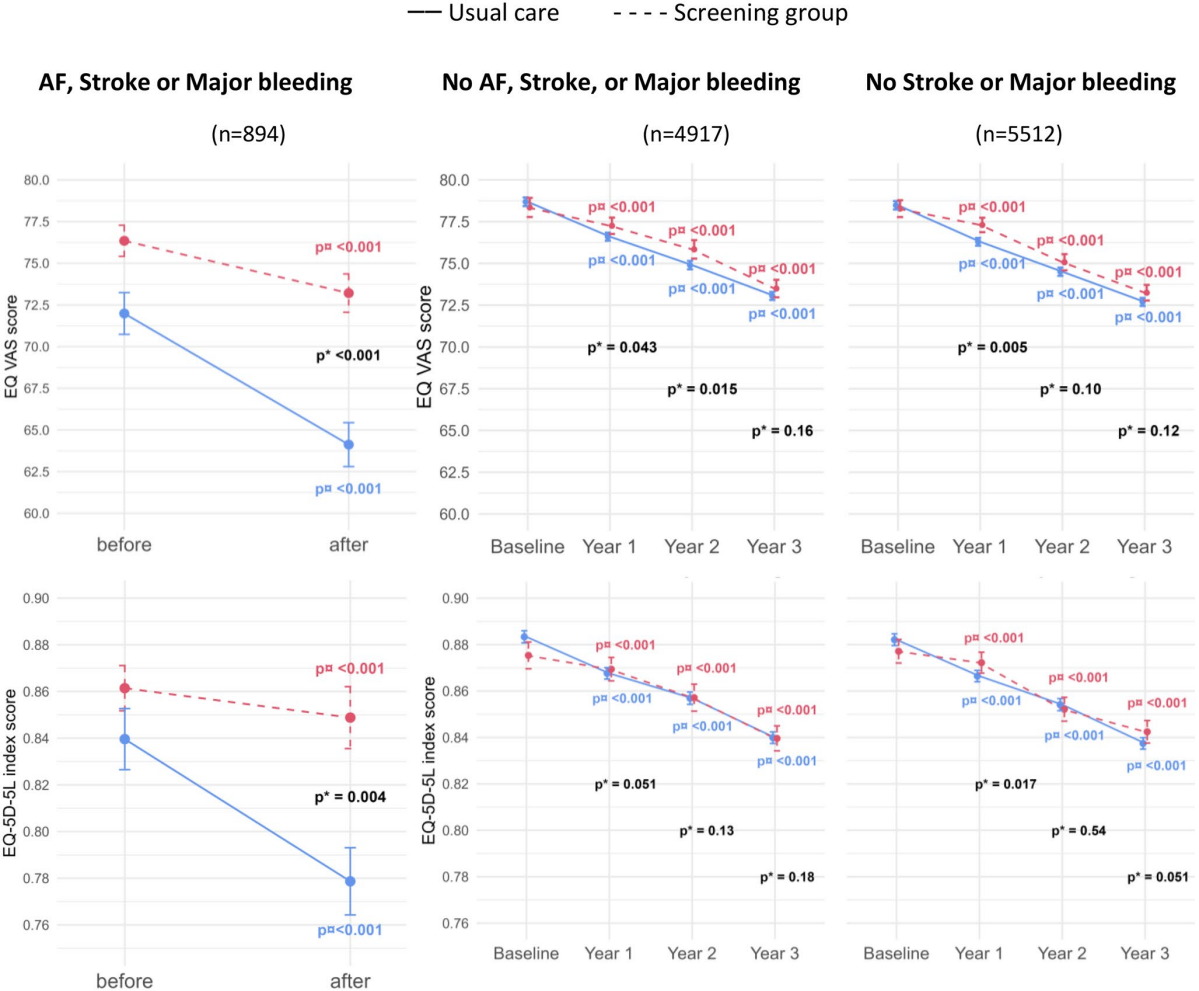
Quality of life in participants alive until year three. Abbreviations: AF: Atrial fibrillation. *p for difference in decline between screening and usual care (interaction analyses). p¤ for difference in HRQoL from baseline until year three within randomisation group

The combined outcome of AF, stroke and major bleeding resulted in an EQ-VAS score decline from 76.3 before to 73.2 after diagnosis (*p* < 0.001) in the screening group and from 72.3 before to 64.1 after diagnosis (*p* < 0.001) in usual care, with 4.28 (CI: 1.74;6.82) more decline in the usual care group than in the screening group (*p* < 0.001). The EQ-5D-5L index score declined from 0.86 before to 0.85 after diagnosis (*p* < 0.001) in the screening group and from 0.84 before to 0.78 after diagnosis (*p* < 0.001) in usual care, which resulted in 0.048 (CI: 0.02;0.077) more decline in the usual care group than in the screening group (*p* < 0.001) (Fig. 3).

### HRQoL after freedom from AF, stroke and major bleeding

During three years, 4917 participants evaded both AF, stroke and major bleeding, 1008 (20%) in the screening group and 3909 (79.5%) in usual care, respectively. From baseline to the end of year three, the EQ-VAS score declined from 78.4 to 73.5 (*p* < 0.001) in the screening group and from 78.7 to 73.1 (*p* < 0.001) in usual care, with a higher score in the screening group at the one-year and two-year assessment (one-year difference: 0.99 (CI: 0.03;1.95, *p* = 0.04), two-year difference: 1.28 (CI: 0.25;2.31, *p* = 0.015)), but not at the three-year assessment compared to usual care (Fig. 3). The EQ-5D-5L index score declined from 0.88 at baseline to 0.84 at the end of year three (*p* < 0.001) in both randomisation groups, with a higher score in the screening group at the one-year assessment (difference: 0.01 (CI: <0.001;0.02, *p* = 0.051)), but not at the two-year and three-year assessments compared to usual care. The annual HRQoL changes in participants free of AF, stroke and major bleeding are listed in Supplementary Table 1.

### Major problem in life domains

The occurrences of any major problem did not differ after AF between the randomisation groups (1.25 (CI: 0.88;1.79, *p* = 0.20) compared to before, in all five EQ-5D-5L domains. For participants diagnosed with stroke, or major bleeding, or the combination of AF or stroke or major bleeding, the occurrences of any major problem did not significantly differ either (OR 1.3 (CI: 0.53;3.12, *p* = 0.56), OR 0.62 (CI: 0.24;1.57, *p* = 0.31), and OR 0.93 (CI: 0.67;1.28, *p* = 0.63)). The occurrence of major problem in each domain at year three was similar between the randomisation groups (Supplementary Fig. S2).

### Prior stroke and new events

A total of 1509 (25%) participants had prior stroke at baseline, with 1417 alive at year three, and there were no HRQoL differences between the screening group and usual care at the three-year assessment. In participants with prior stroke who were diagnosed with a combination of either AF, incident stroke or major bleeding, there was no difference in EQ-VAS score between the randomisation groups before and after detection (*p* = 0.32), whereas the EQ-5D-5L index score declined from 0.86 before to 84 after diagnosis in the screening group, and from 0.84 before to 0.76 after diagnosis in usual care, with less HRQoL decline in the screening group (*p* = 0.02). In participants with prior stroke, and diagnosed with AF, incident stroke or major bleeding, there were no HRQoL differences between the randomisation groups.

### Sex and age

At the three-year assessment, male participants had higher HRQoL (EQ-VAS score difference: 2.66 (CI 1.7;3.6; *p* < 0.001), EQ-5D-5L index score difference: 0.04 (CI: 0.03;0.05, *p* < 0.001)), and males diagnosed with AF during three years had higher EQ-5D-5L index scores compared to females diagnosed with AF (difference: 0.06 (CI: 0.01;0.1, *p* = 0.015)). At the three-year assessment, older participants (aged 75–90) had lower HRQoL (EQ-VAS score difference: -1.97 (CI: -3.00; -0.96, *p* = 0.0001), EQ-5D-5L index score difference: -0.02 (CI: -0.03;-0.01, *p* = 0.001)) compared to younger participants (aged 70–74). Older participants diagnosed with AF had similar HRQoL compared to younger participants diagnosed with AF.

## Discussion

This was a post-hoc analysis of the LOOP study on HRQoL, based on 6004 participants who were randomised to usual care or continuous heart rhythm monitoring with ILR and anticoagulation upon AF detection. Our main findings were (1) AF diagnoses were generally followed by minor or moderate HRQoL reductions, while we found large reductions after stroke and major bleeding, compared with HRQoL before these diagnoses, (2) For participants diagnosed with AF, we found different HRQoL changes in the two randomisation groups. AF detected by screening seemed to elicit a minimal negative impact on HRQoL compared with clinical AF, regardless of the time of diagnosis in the study. (3) For participants free of AF, stroke and major bleeding, screening was associated with less decline in HRQoL compared to usual care.

### The impact of AF, stroke and major bleeding on HRQoL

AF patients constitute a heterogeneous population with regard to non-modifiable characteristics, comorbidities, and risk of complications. These differences are also reflected in the two AF types we investigated in our substudy, which compared device-detected AF from screening, diagnosis and during treatment with clinical AF detected by a 12-lead ECG. The full distinction between these AF types and the related risks and outcomes is not yet clear, and more studies are needed to illuminate the differences between them. This is the first study reporting on HRQoL associated with AF, stroke, and major bleeding after continuous population-based AF screening, which allowed a longitudinal investigation of HRQoL between screening-detected AF and conventionally diagnosed AF.

After events of AF, stroke, and major bleeding, the HRQoL was higher in the screening group compared to usual care. For AF alone, our results show that the HRQoL declined significantly less after screening-detected AF compared to clinical AF, when investigating the closest HRQoL assessments before and after AF diagnosis. The baseline HRQoL in these participants was higher for the screening group compared to those in usual care. This difference was mainly driven by those who were diagnosed in the first year of the study, as the baseline HRQoL was the same for participants diagnosed in year two and three. The difference may partly be explained by the faster and higher AF detection rate in the screening group [7], which also included faster treatment of AF, potentially preventing quality of life changes. When we investigated HRQoL in participants who remained alive for all three years, the HRQoL difference after AF detection was even more pronounced between the screening group and usual care, and regardless of the year of AF detection, the screening group had higher HRQoL than usual care. When compared to AF-free participants, HRQoL was not reduced at year three in participants who were diagnosed and treated for AF through screening. For participants with conventionally diagnosed and treated AF, the HRQoL had declined twofold compared to AF-free participants at the three-year assessment. These analyses may include the healthiest participants with higher HRQoL, while excluding those who might have reported worse HRQoL scores before death. Even so, any population-based screening strategy for AF may likely include many healthy participants and fewer very ill participants. In the entire LOOP population, the HRQoL during three years was not different in screened participants vs. in usual care [16]. The analyses of AF, stroke, or major bleeding combined support that the overall impact of AF screening, when including the arguably most important clinical events related to AF screening, does lead to a higher HRQoL compared to the same outcomes diagnosed and treated in usual care.

Anxiety has been linked with both screening itself and with diagnoses of AF [4, 5, 17, 18]. We found HRQoL improvements in the screening group, but these were not accompanied by a separate reduction in anxiety/depression or improvements in the other life domains either. Further, we found larger EQ-VAS differences between the randomisation groups than with the EQ-5D-5L index scores. Still, our results may reflect a true HRQoL increment in the screening group, as the HRQoL scores are often not aligned [10]. The EQ-VAS score is strongly associated with the scores in the EQ-5D-5L domains, but tends to be more dynamic and may better reflect participants’ overall health, especially when changes occur in dimensions outside the predefined descriptive system of the EQ-5D-5L index [10]. This can result in EQ-5D-5L index scores that remain unchanged over time, while the EQ-VAS changes simultaneously, as has been reported particularly in elderly populations [10, 19]. As the intervention in this study was unblinded, the differences in EQ-VAS scores between the screening group and usual care could reflect feelings of reassurance or safety, which are not measured in the EQ-5D-5L index [19]. All baseline HRQoL assessments were performed before randomisation, which supports that screening-detected AF is associated with higher HRQoL than conventionally diagnosed AF.

The EQ-5D-5L index can be used to measure HRQoL in stroke patients in the rehabilitation period [20–22], and we found no difference in HRQoL scores after stroke between the randomisation groups. We found no difference in median time to stroke between the groups, and our results were robust to adjustment for age, stroke severity, stroke type, and AF diagnosis. For screened participants with prior stroke, the EQ-5D-5L index score was slightly higher compared to usual care after AF, stroke or major bleeding, with a trend towards maintained difference in the following assessment. This finding is in line with another LOOP analysis of stroke severity, where screening was associated with a lower risk of severe stroke, however this was among participants without prior stroke [20]. 

### The relationship between screening and freedom of events

Participants free of AF, stroke, or major bleeding seemed to have higher short-term HRQoL in the screening group than in usual care (Fig. 2, 3, Supplementary Table 1). These differences were mostly found in the EQ-VAS score. This could indicate true increments in health-related quality of life or be interpreted merely as a sense of relief or reassurance associated with negative findings related to screening. Yet, significant differences were also found in the EQ-5D-5L index scores when compared to usual care , further indicating that, also in the absence of events, screening may improve HRQoL in the short term compared to usual care. In participants free of stroke and major bleeding, we found screening to be associated with a small benefit in the EQ-5D-5L index score at year three. We investigated the separate life domains, but did not detect any differences between the groups.

### Clinical perspectives

Intensive screening detects AF in up to 30% of an at-risk population, which seems associated with a lower stroke risk compared to atrial fibrillation detected by usual care (e.g. with a 12-lead ECG) [21–24]. Nonetheless, screening is increasing. We have presented novel data from one of the largest randomised trials investigating one adverse effect of AF screening. Our findings suggest that quality of life is not impaired by screening, which was also reported in the DANCAVAS trial, which investigated population-based screening for cardiovascular disease [3]. In contrast, the SAFE trial reported higher anxiety scores after non-continuous AF screening and diagnoses, though this was compared to AF-free participants in the intervention arms [25]. Further data on the psychological impact are also planned in the ongoing SAFER trial, which investigates daily handheld ECG screening vs. control [26]. Understanding treatment-related changes in HRQoL is important for both patients and physicians, as screening should provide an overall net benefit to the patient. HRQoL has been associated with the risks of death and hospitalisation [11, 28], and patients may often overestimate the benefit of screening and treatment, while underestimating the associated harms [27]. We found female sex and higher age generally associated with a decreasing HRQoL, which corresponds to other studies on population health and underlines the likely differentiated screening impact in selected populations [19, 28]. While further randomised trials are needed to confirm the psychological and health-related impact of detecting arrhythmia through continuous heart rhythm screening, we believe our results support the current screening strategy in the European AF guidelines, since HRQoL does not seem to be harmed by intensive screening [2]. Further, detection of AF may hold the potential of improving health in other outcomes [29]. Lastly, the opportunities for screening have increased beyond professional healthcare settings, suggesting that consumer-led and clinician-led screening will likely coexist in the years to come. While our results are not directly comparable with those from wearables (e.g., smartwatches), they may provide insight into the impact of continuous screening on HRQoL in general.

### Limitations

This was a study with post-hoc analyses and exploratory outcomes, which inherently increases the risk of type one errors and spurious findings, and the results should be hypothesis-generating. In the control group, HRQoL data were collected by letter at year one and year two, which could have introduced sampling bias since these time points had more missing data than baseline and year three. Furthermore, HRQoL data can change over short periods and be affected by factors not measured in the study, rendering interpretation difficult. The EQ-5D-5L questionnaire provides generic assessments of quality of life, which may overlook changes outside the predefined dimensions. We included all visits in our primary analyses using advanced linear mixed models and used sensitivity analyses to adjust for confounding parameters. Incomplete data not attributed to death were addressed as missing at random, and were estimated through correlations from other baseline variables. Lastly, a healthy-user bias may occur in the trial population, since letter recruitment may exclude those with more illnesses while including those who tend to engage in disease-preventing measures or activities.

## Conclusion

In this trial with high-risk persons randomised to AF screening or usual care, screening-detected AF had a low impact on quality of life compared to conventionally diagnosed AF, which was associated with a two-fold larger HRQoL decline over three years compared to AF-free participants. Stroke and major bleeding were both associated with a considerable negative impact on HRQoL, regardless of randomisation group. Participants free of AF, stroke and major bleeding had less decline in HRQoL in the screening group compared to usual care, indicating that negative screening (without findings) may be associated with beneficial outcomes.

## Supplementary Information

Below is the link to the electronic supplementary material.

Supplementary Material 1

## Abbreviations

AF: Atrial fibrillation
CI: Confidence interval
COPD: Chronic obstructive pulmonary disease
ECG: Electrocardiogram
EQ-VAS: EuroQol visual analogue scale
EQ-5D-5L: EuroQol 5D-5 L
HRQoL: Health-related quality of life
ILR: Implantable loop recorder
TIA: Transient ischemic attack
OR: Odds ratio
SD: Standard deviation
SAE: Systemic arterial embolism

## Declarations

### Data sharing statement

The study data underlying this article cannot be shared publicly for ethical reasons, but the methodology will be shared on reasonable request to the corresponding author (SZD).
