# Supplementary material for: The impact of screening-detected atrial fibrillation and associated outcomes on quality of life
Source: Qual Life Res. 2025 Aug 31;34(12):3597–608. doi: 10.1007/s11136-025-04047-1 (PMC12689788; doi:10.1007/s11136-025-04047-1)
Supplement: Supplementary file 1 — Supplementary Material 1 [file 11136_2025_4047_MOESM1_ESM.docx]

**SUPPLEMENTARY DATA**

**Supplementary Table 1**

**Annual quality of life estimates in participants without atrial fibrillation, stroke or major bleeding**

|  |  | **Year one** | | | **Year two** | | | **Year three** | | |
| --- | --- | --- | --- | --- | --- | --- | --- | --- | --- | --- |
|  |  |  |  |  |  |  |  |  |  |  |
| *Groups* | *Outcomes* | *n (%)* | *HRQoL*  *Difference** | *p* | *n (%)* | *HRQoL Difference** | *p* | *n (%)* | *HRQoL Difference** | *p* |
| **No AF** | EQ-VAS | 5,626 (94%) | 1.26 (0.34;2.18) | **0.007** | 5358 (89%) | 1.18 (0.18;2.19) | **0.02** | 5097 (85%) | 0.85 (-0.23;1.93) | 0.12 |
|  | EQ-5D-5L |  | 0.008 (-0.002;0.02) | 0.11 |  | 0.005 (-0.005;0.015) | 0.34 |  | -0.007 (-0.02;0.004) | 0.21 |
| **No Stroke** | EQ-VAS | 5,894 (98%) | 1.35 (0.49;2.21) | **0.002** | 5746 (96%) | 0.62 (-0.28;1.52) | 0.18 | 5589  (93%) | 0.75 (-0.19;1.68) | 0.12 |
|  | EQ-5D-5L |  | 0.01 (0.002;0.02 | **0.02** |  | 0.001 (-0.008;0.11) | 0.82 |  | 0.01 (<0.001;0.02)) | **0.04** |
| **No Major bleeding** | EQ-VAS | 5,911  (98%) | 1.4 (0.51;2.22) | **0.002** | 5776 (96%) | 1.2 (0.40;2.07) | 0.13 | 5634 (94%) | 0.83 (-0.11;177) | 0.08 |
|  | EQ-5D-5L |  | 0.01 (0.002;0.02) | **0.02** |  | 0.004 (-0.006;0.01) | 0.45 |  | 0.01 (0.002;0.02) | **0.04** |

*Difference between the screening group and usual care (ref= usual care)

Abbreviation: HRQoL: Health-related quality of life, AF: Atrial fibrillation, EQ VAS: EuroQol visual analogue scale, EQ-5D-5L: EuroQol 5D-5L

**Supplementary Figure 1**

**Quality of life after Atrial fibrillation diagnosis grouped by year of detection**

**
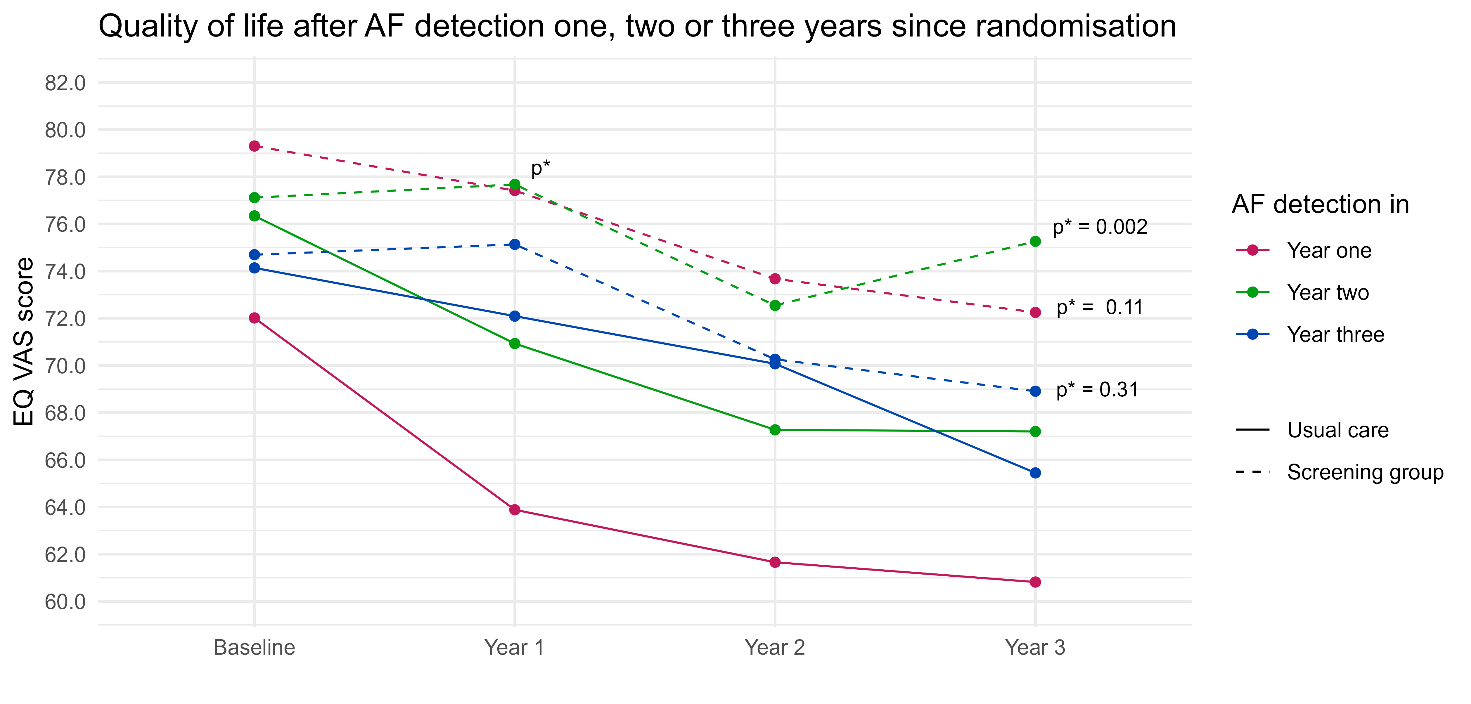
**

n=302 n=173 n=161

**
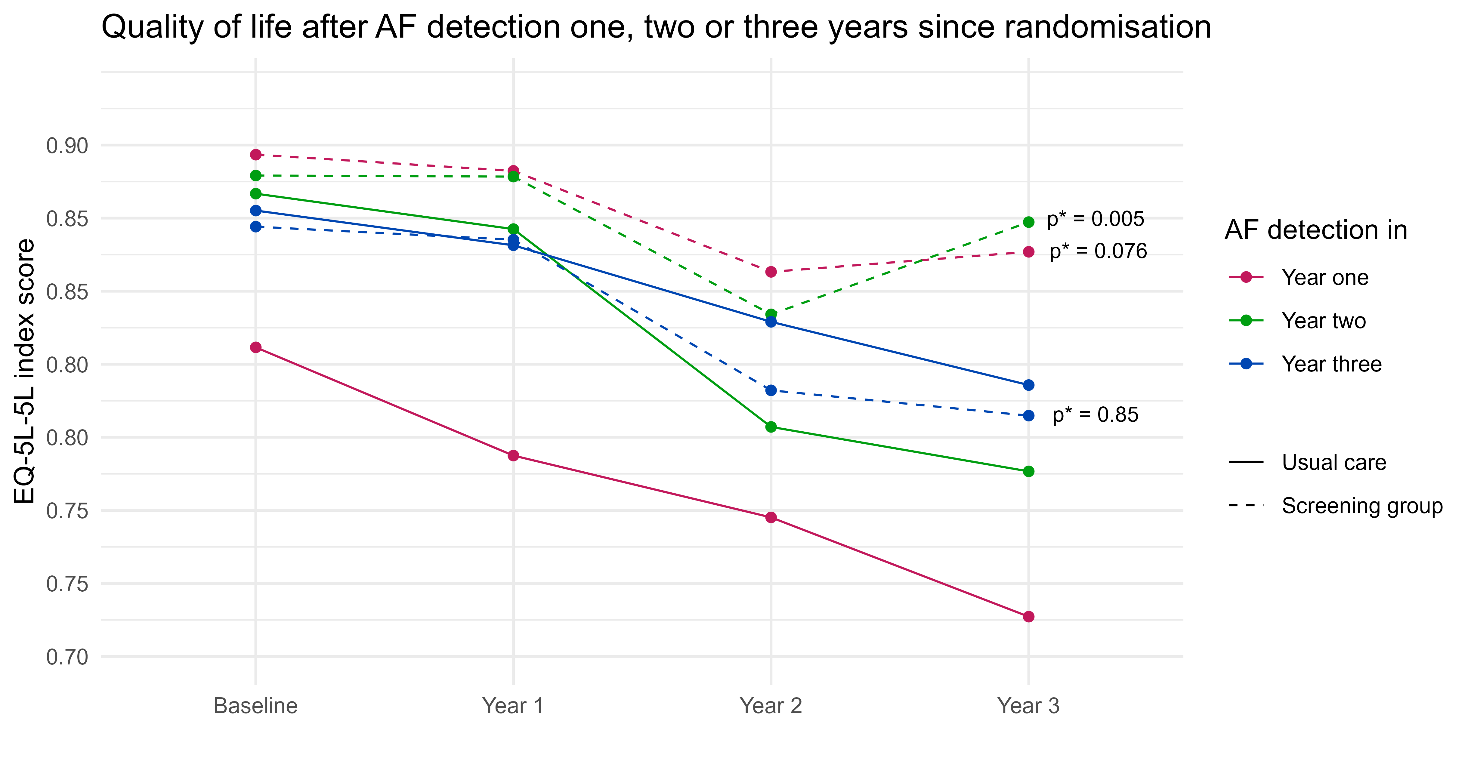
**

n=302 n=173 n=161

Assessments

n= number of annual AF events in participants alive

p* for difference in HRQoL decline between screening and usual care (interaction analysis)

p* values were 0.01 and 0.018 in EQ-VAS for AF detection in year one and two, respectively (interaction analyses)

All HRQoL reductions from baseline within randomisation groups were statistically significant.

**Supplementary Figure 2**

**Major problem after events in the screening arm compared to usual care**

**
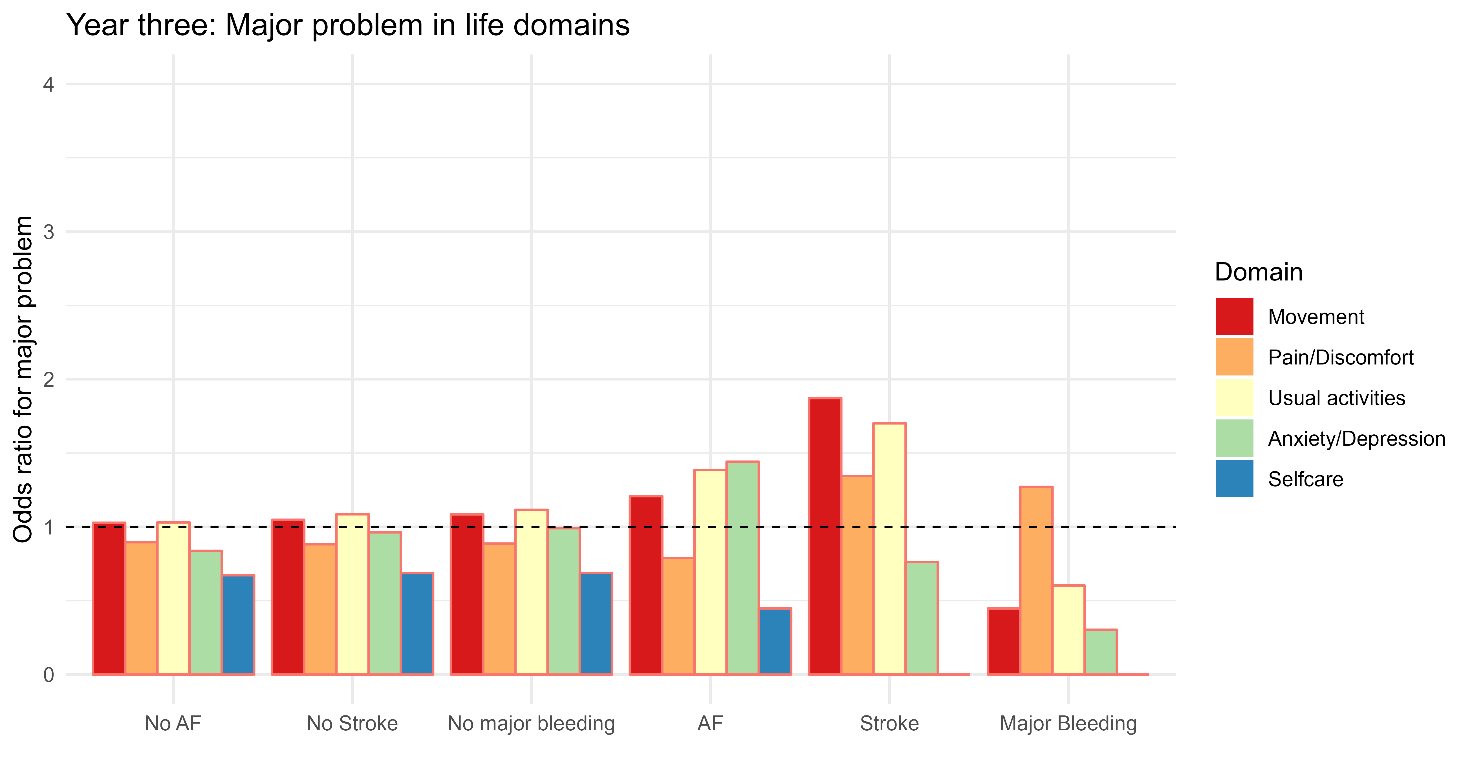
**

**
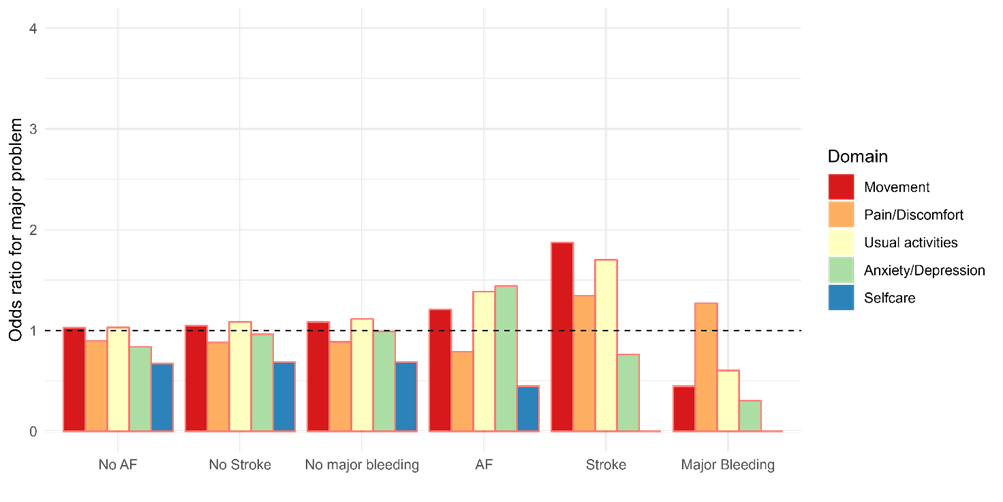
**

Estimates above 1.00 favour usual care, and estimates below 1.00 favour screening.

*None of the odds ratio estimates were statistically significant
